# Stimulus strength determines the BTK-dependence of the SHIP1-deficient phenotype in IgE/antigen-triggered mast cells

**DOI:** 10.1038/s41598-018-33769-1

**Published:** 2018-10-19

**Authors:** Carolin N. Zorn, Anne Simonowski, Michael Huber

**Affiliations:** Institute of Biochemistry and Molecular Immunology, Faculty of Medicine, RWTH Aachen University, Pauwelsstraße 30, 52074 Aachen, Germany

## Abstract

Antigen (Ag)-mediated crosslinking of IgE-loaded high-affinity receptors for IgE (FcεRI) on mast cells (MCs) triggers activation of proinflammatory effector functions relevant for IgE-associated allergic disorders. The cytosolic tyrosine kinase BTK and the SH2-containing inositol-5′-phosphatase SHIP1 are central positive and negative regulators of Ag-triggered MC activation, respectively, contrarily controlling Ca^2+^ mobilisation, degranulation, and cytokine production. Using genetic and pharmacological techniques, we examined whether BTK activation in *Ship1*−/− MCs is mandatory for the manifestation of the well-known hyperactive phenotype of *Ship1*−/− MCs. We demonstrate the prominence of BTK for the *Ship1*−/− phenotype in a manner strictly dependent on the strength of the initial Ag stimulus; particular importance for BTK was identified in *Ship1*−/− bone marrow-derived MCs in response to stimulation with suboptimal Ag concentrations. With respect to MAPK activation, BTK showed particular importance at suboptimal Ag concentrations, allowing for an analogous-to-digital switch resulting in full activation of ERK1/2 already at low Ag concentrations. Our data allow for a more precise definition of the role of BTK in FcεRI-mediated signal transduction and effector function in MCs. Moreover, they suggest that reduced activation or curtate expression of SHIP1 can be compensated by pharmacological inhibition of BTK and *vice versa*.

## Introduction

Mast cells (MCs) are critical effector cells in IgE-associated allergic disorders^1^. Antigen (Ag)-mediated crosslinking of IgE-loaded high-affinity receptors for IgE (FcεRI) on MCs drives immediate secretion of preformed mediators (e.g. biogenic amines and proteases) and de-novo synthesis of proinflammatory mediators (e.g. leukotrienes and cytokines)^1^. MC activation is regulated by complex signalling networks^2^. One crucial signalling element downstream of the FcεRI is the phosphatidylinositol-3-kinase (PI3K) phosphorylating phosphatidylinositol-4,5-bisphosphate (PI-4,5-P_2_) to yield the second messenger phosphatidylinositol-3,4,5-trisphosphate (PIP_3_). Several central signalling proteins contain pleckstrin homology (PH)-domains allowing them to bind to PIP_3_. Amongst these are PKB and Bruton’s tyrosine kinase (BTK)^3^.

Binding of PIP_3_ to its N-terminal PH-domain appears to release BTK from an auto-inhibited conformation, allowing transphosphorylation by LYN at Y551 in BTK’s kinase domain^4,5^. This results in activation of BTK, which then autophosphorylates at Y223 within its SH3-domain^6^. Though Y223 phosphorylation has only a minor role in regulating BTK catalytic activity^7^, it serves as an early substrate for activated BTK and thus, it can serve as indirect measure for BTK activity. By phosphorylating phospholipase C (PLC)γ, BTK controls Ag-triggered Ca^2+^ mobilisation, and hence, *Btk*−/− MCs hardly mobilise Ca^2+^ in response to Ag^8,9^. Moreover, BTK positively regulates Ag-induced cytokine production in MCs^10^.

In MCs, a crucial negative regulator of the PI3K pathway is the SH2-containing inositol-5′-phosphatase 1 (SHIP1), which dephosphorylates PIP_3_ generating PI-3,4-P_2_^11^. *Ship1*−/− bone marrow-derived MCs (BMMCs) degranulate much stronger in response to Ag than wild-type (WT) BMMCs, which goes along with augmented store-operated Ca^2+^ entry^12,13^. Moreover, SHIP1 suppresses Ag-triggered degranulation in response to high, so-called supra-optimal Ag concentrations, contributing to the well-known bell-shaped dose-response curve of MC activation^14,15^. Furthermore, SHIP1 represses Ag-induced proinflammatory cytokine production^16,17^.

Interestingly, MC functions upregulated in *Ship1*−/− BMMCs (e.g. Ca^2+^ mobilisation, degranulation, and cytokine production)^12,16,17^ are suppressed in *Btk*−/− BMMCs^10,18^. Moreover, BTK activation appears to depend on the SHIP1 substrate PIP_3_. Hence, BTK often is discussed as a central mediator of the hyperactive *Ship1*−/− effector phenotype. However, there are also studies challenging the strict PI3K-dependence of BTK activation^15,19^. As mentioned earlier, SHIP1 is activated in particular after stimulation with supra-optimal Ag concentrations resulting in reduced PI3K-dependent PKB phosphorylation compared to stimulation with optimal Ag concentrations^15^. Phosphorylation of BTK at Y223, however, was not found to be attenuated after stimulation with supra-optimal Ag doses^15^. Moreover, Suzuki *et al*. showed in B-lymphocytes that activation of the IgM B-cell Ag receptor resulted in BTK phosphorylation that was not reduced in the presence of PI3K inhibitors^19^. In addition, BTK phosphorylation was even enhanced in B-lymphocytes deficient for the regulatory PI3K subunit, p85α^19^. These data suggested that the functional relationship between PI3K and BTK might depend on the receptor/ligand system studied and the cell type investigated.

In the present study, we analysed the PI3K-dependence of BTK function in more detail. Using BMMCs double-deficient for SHIP1 and BTK (from now on called DKO), we examined whether BTK activation in *Ship1*−/− BMMCs is mandatory for the manifestation of the various hyperactive phenotypes of *Ship1*−/− BMMCs. We show that BTK is phosphorylated at Y223 and Y551 in MCs independent of PI3K activation. Moreover, using DKO BMMCs we demonstrate the importance of BTK for the *Ship1*−/− phenotype in a manner strictly dependent on the strength of the initial Ag stimulus; particular importance for BTK was identified in response to stimulation with low (suboptimal) Ag concentrations. Furthermore, our data indicate that the digital nature of MAPK ERK1/2 activation in BMMCs is dependent on BTK. Our data allow for a more precise definition of the role of BTK in FcεRI-mediated signal transduction and effector functions in MCs.

## Results

### PI3K-independent phosphorylation of BTK and PLCγ1 in Ag-stimulated murine MCs

**Figure 1:**
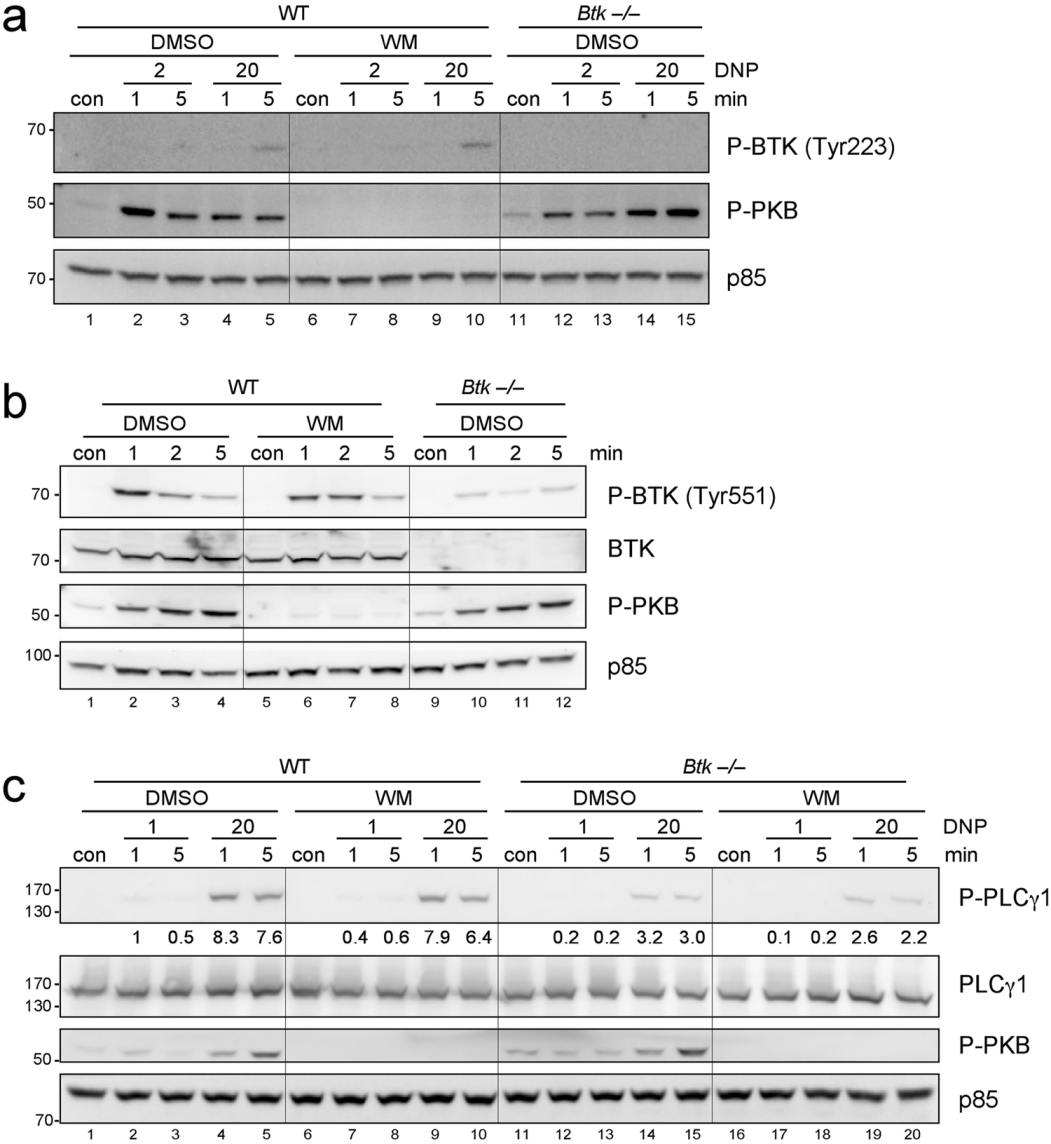
Antigen-triggered phosphorylation of BTK and PLCγ in BMMCs is independent of PI3K. (**a**) WT BMMCs were pre-treated for 20 min with 100 nM Wortmannin (WM) or vehicle (DMSO) and were then left untreated (con) or stimulated with the indicated concentrations of Ag (DNP-HSA [DNP]) for 1 and 5 min. *Btk*−/− BMMCs were pre-treated with DMSO and stimulated accordingly. Whole-cell lysates were subjected to Western Blot analysis with antibodies against P-BTK (Tyr223), P-PKB (Ser473), and p85 (loading control). (**b**) WT BMMCs were pre-treated for 20 min with 100 nM WM or vehicle (DMSO) and were then left untreated (con) or stimulated with Ag (20 ng/ml DNP-HSA) for the indicated times. *Btk*−/− BMMCs were pre-treated with DMSO and stimulated accordingly. Whole-cell lysates were subjected to Western Blot analysis with antibodies against P-BTK (Tyr551), BTK, P-PKB (Ser473), and p85 (loading control). (**c**) WT and *Btk*−/− BMMCs were pre-treated for 20 min with 100 nM WM or vehicle (DMSO) and were then left untreated (con) or stimulated with the indicated concentrations of Ag for 1 and 5 min. Whole-cell lysates were analysed by immunoblotting against P-PLCγ1 (Tyr783), PLCγ1, P-PKB (Ser473), and p85 (loading control). Densitometry of P-PLCγ1 with reference to PLCγ1 was performed using ImageJ software (NIH, USA) and normalised to WT cells pretreated with DMSO and stimulated with 1 ng/ml Ag (1 min). Comparable results were obtained in at least three experiments with different BMMC cultures. Fine vertical lines were inserted to allow for better discrimination of cells/conditions.

In previous work, we and others have shown that SHIP1-dependent hydrolysis of PIP_3_ is particularly important in regulating responses to FcεRI crosslinking by supra-optimal Ag concentrations^14,15,17,20^. This also suggested that BTK, due to its reported PI3K-dependence, is involved in controlling the hyperactive *Ship1*−/− phenotype. However, BTK activation was also shown to be independent on PI3K, using genetic and pharmacological approaches in B cells^19^. Thus, we re-addressed the PI3K-dependence of BTK phosphorylation in Ag-stimulated MCs. IgE-loaded BMMCs were stimulated with two doses of the Ag DNP-HSA (suboptimal: 2 ng/ml; optimal: 20 ng/ml) in the presence or absence of the pan-specific PI3K inhibitor Wortmannin and activating phosphorylations of BTK (at Y223) and PKB (at S473) were analysed. Whereas PKB phosphorylation was completely inhibited by Wortmannin, BTK phosphorylation appeared unaltered (Fig. 1a), suggesting that BTK-dependent signalling events can be independent of PI3K. To control for specificity of the anti-P-BTK antibody, *Btk*−/− BMMCs were included (Fig. 1a).

However, since phosphorylation of BTK at Y223 can also occur in a BTK-independent manner^21,22^, we additionally analysed phosphorylation of Y551 of BTK in response to Ag (20 ng/ml) in BMMCs pre-treated with DMSO or 100 nM Wortmannin. Cells were left unstimulated or stimulated for 1, 2, and 5 min. In addition, DMSO-pre-treated *Btk*−/− BMMCs were stimulated with Ag. Control Western blots with BTK-, p85-, and P-PKB (Ser473)-specific antibodies corroborated lack of BTK in *Btk*−/− BMMCs, equal loading, and efficiency of PI3K inhibition, respectively (Fig. 1b). The analysis using a phospho-Y551-specific antibody revealed fast phosphorylation (within 1 min) upon Ag treatment with a subsequent decline in intensity, indicating that phosphorylation of Y551 precedes phosphorylation of Y223 (Fig. 1b). Phosphorylation of Y551 upon Ag stimulation was weakly attenuated by PI3K inhibition. Unexpectedly, a signal was obtained even in *Btk*−/− BMMCs. Further analysis revealed that this protein migrated faster compared to BTK. Moreover, using the BLAST tool, a high homology was found between the TEC family kinases BTK, TEC, ITK, and TXK (which are all expressed in BMMCs) with respect to the amino acid sequence surrounding Y551 of BTK (Supplementary Fig. S1), suggesting that the phospho-Y551-specific antibody is able to detect different phosphorylated TEC family kinases, particularly in the absence of its high-affinity target BTK. Additionally, cross-reactivity with another phosphorylated protein cannot be ruled out completely.

FcεRI-mediated PLCγ1 phosphorylation has been demonstrated to be independent of PI3K^23^, though dependent on BTK^8,9^. However, others have reported no effect on PLCγ1 phosphorylation in *Btk*−/− BMMCs^10^ and thus we re-addressed this question in our cellular setting. Indeed, PLCγ1 phosphorylation in *Btk*−/− BMMCs was markedly reduced compared to WT cells (Fig. 1c). Moreover, only a minor reduction of PLCγ1 phosphorylation by pharmacological PI3K inhibition was found in WT BMMCs (Fig. 1c). Efficiency of PI3K inhibition was proven by loss of PKB phosphorylation (Fig. 1c). In conclusion, BTK phosphorylation/activation and function in BMMCs can occur in a PI3K-independent manner.

### Degranulation of SHIP1-deficient MCs is dependent on BTK particularly at suboptimal Ag concentrations

Whereas *Ship1*−/− MCs show enhanced degranulation, Ca^2+^ mobilisation, and proinflammatory cytokine production after FcεRI stimulation, *Btk*−/− MCs exert reduced responses compared to WT cells^10,12,13,16,24^. Thus, a crucial role of BTK for shaping the *Ship1*−/− phenotype was presumed, though never addressed. In light of our initial data (Fig. 1), we sought to address this issue by using a genetic approach. Provided that BTK is dominant in transducing the effects of augmented PIP_3_ levels in *Ship1*−/− BMMCs, the phenotype of Ag-stimulated DKO BMMCs should be comparable to *Btk*−/− BMMCs. Thus, *Btk*−/− and *Ship1*−/− mice were crossed and bone marrow from respective F2 progeny animals used for the *in vitro* differentiation of WT, *Btk*−/−, *Ship1*−/−, and DKO BMMCs. The differentiation process was comparable in all cultures and the resulting BMMCs expressed equal amounts of FcεRI, KIT, ST2, and CD13 (Supplementary Fig. S2).

**Figure 2:**
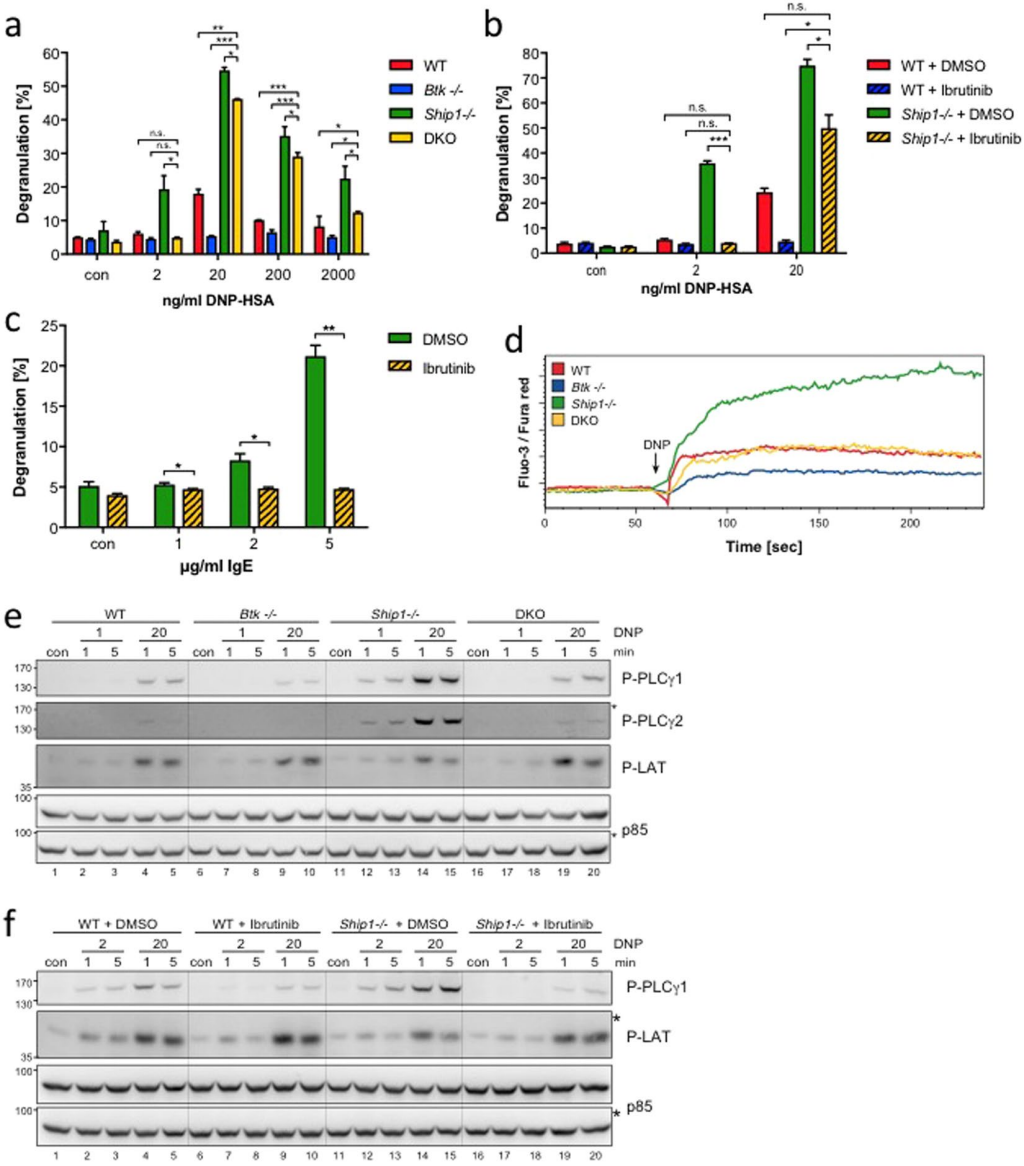
Degranulation of *Ship1*−/− BMMCs in response to suboptimal Ag concentrations is strictly dependent on BTK. (**a**) WT (red), *Btk*−/− (blue), *Ship1*−/− (green), and DKO (yellow) BMMCs were left untreated (con) or stimulated with increasing concentrations of Ag (DNP-HSA) and analysed for the amount of degranulation. Each bar is the mean of duplicates ± SD. (**b**) WT and *Ship1*−/− BMMCs were pre-treated for 30 min with 0.3 µM of the BTK-Inhibitor Ibrutinib or vehicle (DMSO), subsequently left untreated (con) or stimulated with 2 or 20 ng/ml Ag and analysed for the amount of degranulation. Each bar is the mean of triplicates ± SD. (**c**) *Ship1*−/− BMMCs pre-treated with Ibrutinib as in (**b**), were left untreated (con) or stimulated with the indicated amounts of IgE (SPE-7) for the analyses of degranulation. Each bar is the mean of triplicates ± SD. (**d**) Ca^2+^ mobilisation was measured in WT, *Btk*−/−, *Ship1*−/−, and DKO BMMCs upon stimulation with 20 ng/ml DNP-HSA. (**e**) WT, *Btk*−/−, *Ship1*−/−, and DKO BMMCs were stimulated with suboptimal (1 ng/ml) and optimal (20 ng/ml) Ag concentrations for 1 and 5 min or were left untreated (con). Whole-cell lysates were subjected to immunoblot analysis with antibodies against P-PLCγ1 (Tyr783), P-PLCγ2 (Tyr759), P-LAT (Tyr191), and p85 (loading control). (**f**) WT and *Ship1*−/− BMMCs were pre-treated for 30 min with 0.3 µM Ibrutinib or vehicle (DMSO) and then stimulated with the indicated concentrations of Ag. Whole-cell lysates were analysed with antibodies against P-PLCγ1 (Tyr783), P-LAT (Tyr191), and p85 (loading control). Fine vertical lines were inserted to allow for better discrimination of cells/conditions. Because proteins of comparable size were analysed, the same lysates were separated on two gels with an anti-p85 loading control on each gel (marked with/without asterisk). Comparable results were obtained in at least three experiments with different BMMC cultures. Statistical data were analysed for (**a**) n = 6, (**b**) n = 3, and (**c**) n = 3 experiments with n.s. (non significant), *p < 0.05, **p < 0.005, ***p < 0.0005.

First, these BMMCs were analysed with respect to their degranulation capacity. IgE-loaded BMMCs were stimulated with increasing concentrations of Ag and degranulation was measured by means of β-hexosaminidase release. WT BMMCs showed the typical bell-shaped dose-response curve with strongest degranulation at an optimal Ag concentration of 20 ng/ml (Fig. 2a)^15^. As expected, independent of the Ag concentration, *Btk*−/− BMMCs degranulated less or comparably and *Ship1*−/− BMMCs showed augmented secretion (Fig. 2a). DKO BMMCs showed a significant suppression of degranulation compared to *Ship1*−/− cells and degranulated comparably to WT and *Btk*−/− cells in response to a suboptimal Ag concentration (2 ng/ml) (Fig. 2a). At higher Ag concentrations (optimal to supra-optimal), DKO BMMCs degranulated less than Ship1−/− BMMCs, however, significantly stronger than WT BMMCs. This indicated that the quality of the SHIP1-deficient degranulation phenotype, except for suboptimal Ag concentrations, does not strictly depend on expression of BTK.

To corroborate our data from DKO BMMCs, we used a pharmacological approach applying the selective BTK inhibitor, Ibrutinib^25^. Titrating Ibrutinib against Ag-triggered phosphorylation of PLCγ1, 0.3 µM Ibrutinib were determined as sufficient to block BTK-mediated PLCγ1 phosphorylation. WT and *Ship1*−/− BMMCs were then pre-treated with vehicle or 0.3 µM Ibrutinib (to mimic *Btk*−/− and DKO BMMCs, respectively), stimulated with suboptimal and optimal Ag concentrations, and degranulation was measured. Indeed, Ibrutinib-treated WT and *Ship1*−/− BMMCs reacted comparably to *Btk*−/− and DKO BMMCs, respectively (Fig. 2b). Whereas in WT cells, BTK inhibition reduced degranulation independently of the Ag concentration used, Ibrutinib totally prevented degranulation in suboptimally activated *Ship1*−/− BMMCs, however, caused a significant reduction in response to optimal Ag concentration (Fig. 2b).

We showed previously that *Ship1*−/− BMMCs can be induced to degranulate by IgE only, using the cytokinergic IgE, clone SPE-7^12^. Since IgE (SPE-7) is considered to be a weakly FcεRI-crosslinking agent^20^, we expected IgE-triggered degranulation of *Ship1*−/− BMMCs to be strongly suppressed by inhibition of BTK, which was indeed the case (Fig. 2c). In conclusion, our genetic as well as pharmacological approaches demonstrated that the SHIP1-deficient degranulation phenotype of MCs is controlled by BTK in a manner strictly dependent on the extent of FcεRI crosslinking.

### Augmented Ca^2+^ mobilisation in *Ship1*−/− MCs is dependent on BTK

MC degranulation is dependent on store-operated Ca^2+^ entry^26^. Compared to WT cells, *Ship1*−/− and *Btk*−/− BMMCs show augmented and reduced Ca^2+^ mobilisation, respectively^8,9,12^. Thus, we expected from our degranulation results that *Ship1*−/− and DKO BMMCs should not differ severely in Ca^2+^ mobilisation triggered by optimal Ag concentrations. As expected, *Ship1*−/− BMMCs showed a stronger and *Btk*−/− BMMCs a reduced Ca^2+^ flux compared to WT cells. Interestingly, Ca^2+^ mobilisation observed in DKO BMMCs was comparable to WT cells and thus, markedly weaker than in *Ship1*−/− BMMCs (Fig. 2d). This indicated that effects on degranulation and Ca^2+^ mobilisation were quantitatively uncoupled in DKO BMMCs. Such uncoupling has also been reported, amongst others, in *Lyn*-deficient as well as in *Ship1/Lyn* double-deficient BMMCs^27,28^. The exact mechanism has not been elucidated yet.

These Ca^2+^ flux measurements suggested that PLCγ phosphorylation was differentially affected in Ag-triggered *Ship1*−/− and DKO BMMCs. To test this, WT, *Btk*−/−, *Ship1*−/−, and DKO BMMCs were stimulated with suboptimal and optimal Ag concentrations and tyrosine phosphorylation (Y-P) of PLCγ1 and PLCγ2 was measured. In addition, Y-P of the transmembrane adaptor LAT1 was determined, which organises membrane-recruitment of PLCγ. Ag-triggered Y-P of LAT1 appeared to be comparable between the cell types under study (Fig. 2e). In correlation with Fig. 1b, Y-P of PLCγ1 and PLCγ2 was reduced in *Btk*−/− BMMCs compared to WT cells; moreover, Y-P of PLCγ1 and PLCγ2 in *Ship1*−/− BMMCs was drastically augmented (Fig. 2e)^28^. This enhancement was reduced in DKO BMMCs to Y-P levels found in WT cells (Fig. 2e), paralleling the effect on Ca^2+^ mobilisation (Fig. 2d). To corroborate these genetic data, we applied the pharmacological approach using Ibrutinib in WT and *Ship1*−/− BMMCs. Again, LAT phosphorylation appeared comparable in Ag-stimulated WT and *Ship1*−/− BMMCs incubated with DMSO or Ibrutinib (Fig. 2f). PLCγ1 phosphorylation, on the other hand, was reduced in WT and *Ship1*−/− BMMCs pretreated with the BTK inhibitor (Fig. 2f). Thus, our degranulation data indicate a strict BTK-dependence of the *Ship1*−/− phenotype after suboptimal Ag stimulation. BTK dependence of optimally stimulated *Ship1*−/− BMMCs was more pronounced for the regulation of Ca^2+^ mobilisation and preceding signalling events than for degranulation.

### BTK-dependence of cytokine production in *Ship1*−/− MCs is influenced by the extent of FcεRI crosslinking

**Figure 3:**
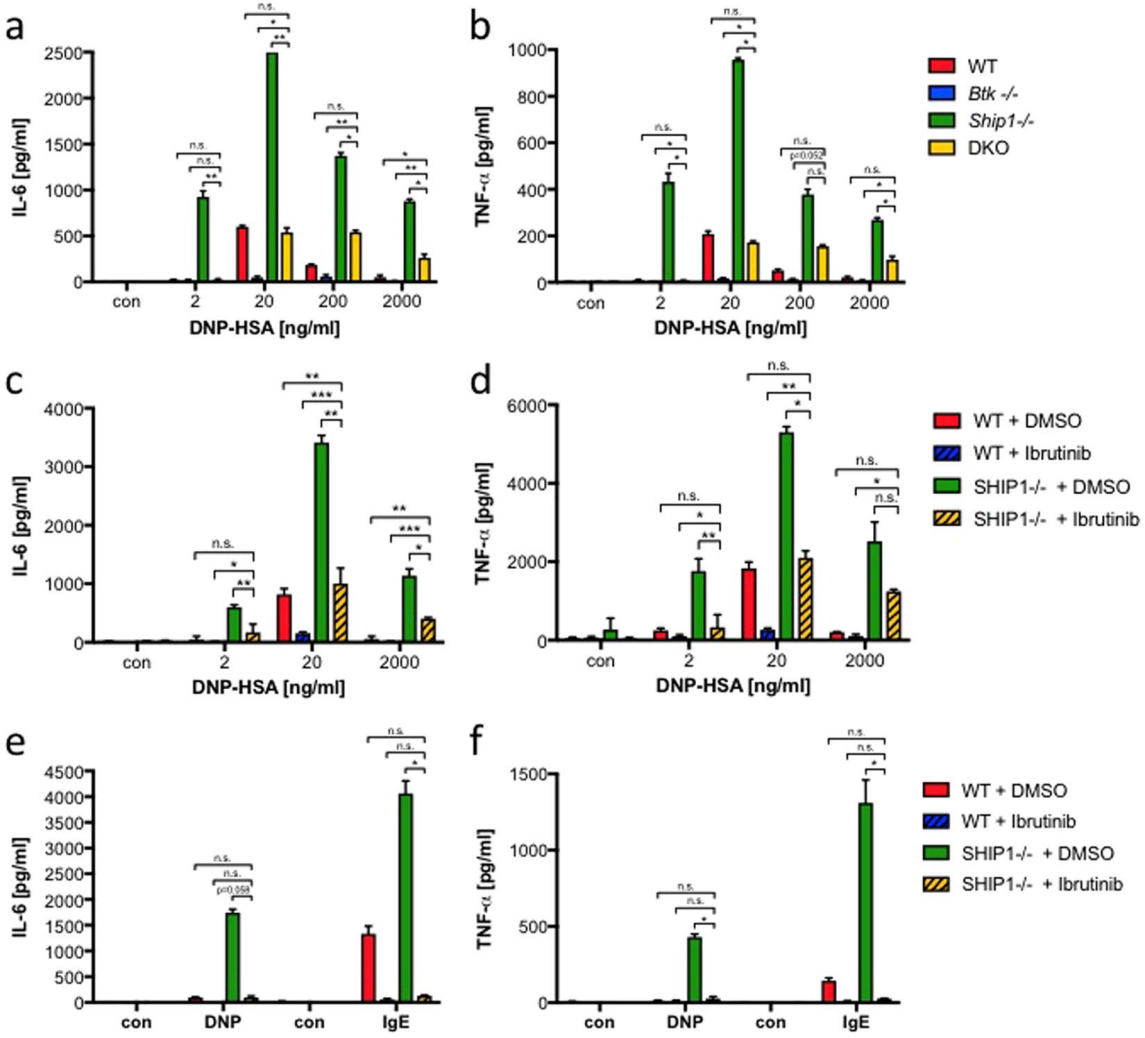
Btk deficiency impacts on Ag-induced cytokine secretion in a dose-dependent manner. (**a**,**b**) WT (red), *Btk*−/− (blue), *Ship1*−/− (green), and DKO (yellow) BMMCs or (**c**,**d**) WT and *Ship1*−/− BMMCs, pre-treated for 30 min with 0.3 µM Ibrutinib or vehicle (DMSO), were left untreated (con) or stimulated with the indicated concentrations of Ag (DNP-HSA). (**e**,**f**) WT and *Ship1*−/− BMMCs were pre-loaded with IgE overnight (for the following DNP-HSA stimulation) or left without IgE (for the following IgE stimulation). Subsequently, the cells were treated for 30 min with 0.3 µM Ibrutinib or vehicle (DMSO). Cells were then left unstimulated (con) or stimulated with Ag (DNP-HSA [DNP]; 2 ng/ml) or with 2 µg/ml IgE (SPE-7). Supernatants were subjected to IL-6 (**a**,**c**,**e**) and TNF-α (**b**,**d**,**f**) ELISAs. Each bar is the mean of triplicates ± SD; comparable results were obtained in at least three experiments with different BMMC cultures. Statistical data were analysed for (**a**) n = 5, (**b**) n = 4, (**c**) n = 6 or n = 4 (2000 ng/ml DNP-HSA), (**d**) n = 5 or n = 3 (2000 ng/ml DNP-HSA), (**e**) and (**f**) n = 3 experiments with n.s. (non significant), *p < 0.05, **p < 0.005, ***p < 0.0005.

Next, we analysed the role of BTK in Ag-triggered production of proinflammatory cytokines in SHIP1-deficient BMMCs. IgE-loaded WT, *Btk*−/−, *Ship1*−/−, and DKO BMMCs were stimulated with increasing concentrations of Ag and IL-6 and TNF-α production was measured. At every Ag concentration, *Ship1*−/− and *Btk*−/− BMMCs produced markedly more and less proinflammatory cytokines than WT BMMCs, respectively (Fig. 3a,b). With respect to DKO BMMCs, again two response patterns dependent on the stimulating Ag dose could be distinguished. In response to suboptimal and optimal Ag concentrations (2 and 20 ng/ml), cytokine production in DKO BMMCs was comparable to WT cells (Fig. 3a,b). At high to supra-optimal Ag concentrations (200 and 2000 ng/ml), DKO cells showed significantly reduced cytokine production compared to *Ship1*−/− cells, however, apparently stronger cytokine secretion than WT cells (Fig. 3a,b). These data corroborated that the SHIP1-deficient phenotype exhibits differential BTK dependencies in an Ag concentration-specific manner.

Moreover, mimicking *Btk*−/− and DKO BMMCs by pretreating WT and *Ship1*−/− BMMCs with Ibrutinib, respectively, the differential importance of BTK activity between cells stimulated with lower and higher Ag doses was verified (Fig. 3c,d). For completion, we combined our pharmacological approach with the weakly crosslinking stimulus, cytokinergic IgE. WT and *Ship1*−/− BMMCs were left untreated or preloaded with IgE overnight. Subsequently, cells were preincubated with vehicle or Ibrutinib and then, BMMCs without prebound IgE were stimulated with IgE alone and IgE-preloaded BMMCs were activated by a suboptimal dose of Ag. Corroborating, BTK inhibition almost completely suppressed IL-6 and TNF-α production in both WT and *Ship1*−/− BMMCs in response to cytokinergic IgE as well as suboptimal Ag concentration (Fig. 3e,f). In conclusion, as observed for the immediate degranulation response, our combined genetic and pharmacological approach showed that in *Ship1*−/− BMMCs, FcεRI-mediated production of proinflammatory cytokines is controlled by BTK in a manner strictly dependent on the extent of receptor crosslinking.

### Signalling defects in BTK-deficient BMMCs were compensated for in BTK/SHIP1 co-deficient cells upon FcεRI aggregation

**Figure 4:**
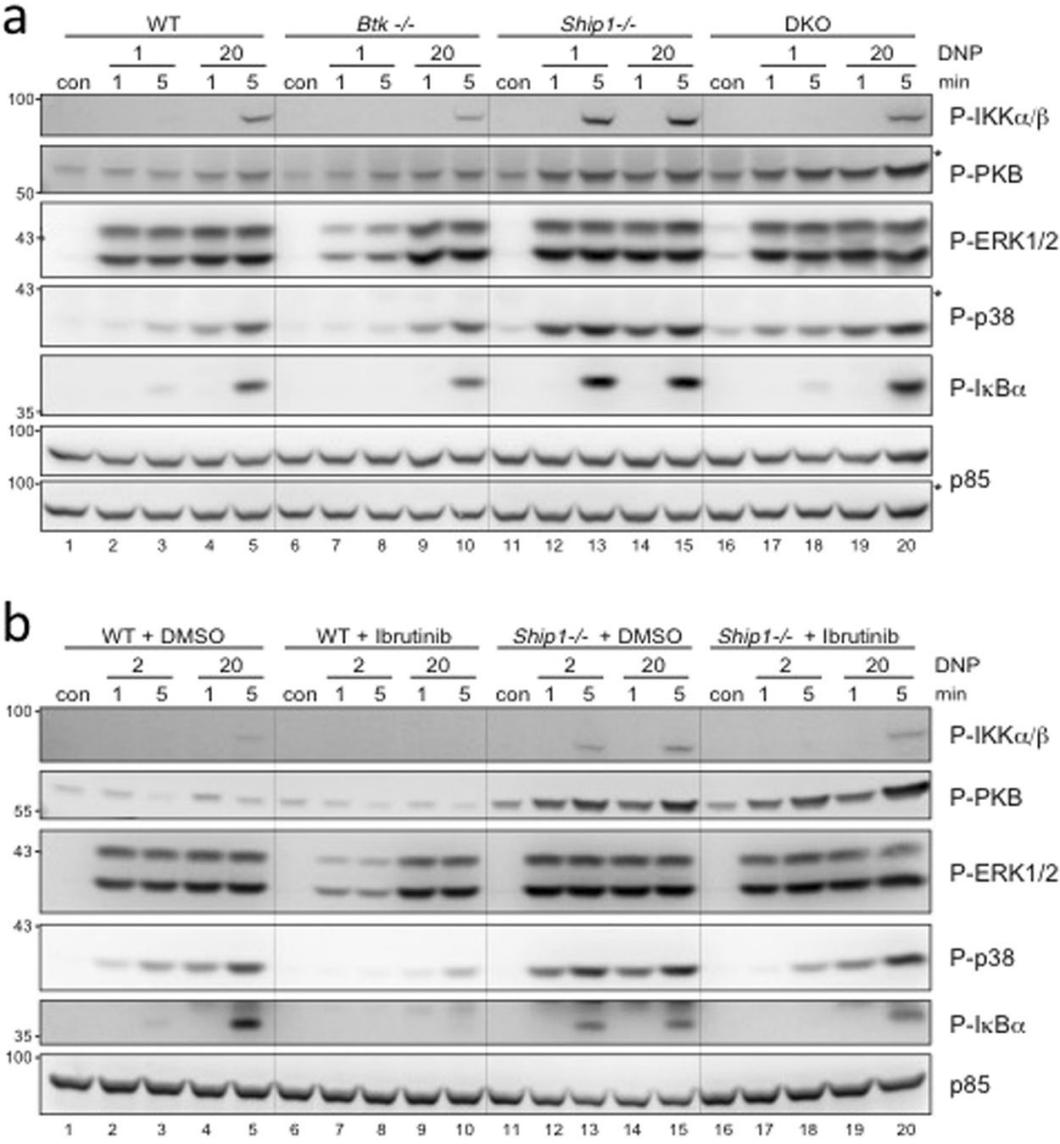
SHIP1-deficiency compensates for signalling defects in BTK-deficient BMMCs. (**a**) WT, *Btk*−/−, *Ship1*−/−, and DKO BMMCs or (**b**) WT and *Ship1*−/− BMMCs, pre-treated with 0.3 µM Ibrutinib or vehicle (DMSO) for 30 min, were left untreated (con) or stimulated with suboptimal and optimal concentrations of Ag (DNP-HSA [DNP]) for 1 and 5 minutes. Whole-cell lysates were subjected to Western Blot analysis with the indicated antibodies against P-IKKα/β (Ser176/Ser177), P-PKB (Ser473), P-ERK1/2 (Thr202/Tyr204), P-p38 (Thr180/Tyr182), P-IκBα (Ser32), and p85 (loading control). Comparable results were obtained in at least three experiments with different BMMC cultures. Fine vertical lines were inserted to allow for better discrimination of cells/conditions. Because proteins of comparable size were analysed, the same lysates were separated on two gels with an anti-p85 loading control on each gel (**a**; marked with/without asterisk).

Subsequently, we analysed activation of signalling molecules involved in the induction of proinflammatory cytokines, namely PKB, IKKα/β, IκBα, and the MAPKs p38 and ERK1/2. IgE-loaded WT, *Btk*−/−, *Ship1*−/−, and DKO BMMCs were stimulated with suboptimal and optimal Ag concentrations for 1 and 5 min and phosphoprotein analysis was performed. The comparison of WT and *Btk*−/− BMMCs stimulated with optimal Ag concentrations (compare lanes 4/5 and 9/10; Fig. 4a) showed reduced phosphorylation of IKKα/β and IκBα in *Btk*−/− cells. As expected^16^, *Ship1*−/− BMMCs showed stronger phosphorylation of PKB, IKKα/β, IκBα, and p38 compared to WT and *Btk*−/− cells independent of the stimulus concentration used (compare lanes 2–5, 7–10, and 12–15; Fig. 4a). Comparing *Ship1*−/− and DKO BMMCs, two response patterns could be distinguished: first, BTK/SHIP1 co-deficiency resulted in reduced p38, IKKα/β, and IκBα phosphorylation compared to *Ship1*−/− BMMCs, particularly in response to suboptimal Ag (compare lanes 12–15 and 17–20; Fig. 4a), indicating BTK-dependence of Ag-induced p38 and IKKα/β activation; second, comparable activation of PKB and ERK1/2 was measured in *Ship1*−/− and DKO BMMCs. Finally, a strong effect was observed for ERK1/2 phosphorylation in response to suboptimal Ag concentrations comparing DKO and *Btk*−/− cells. The marked reduction of ERK1/2 phosphorylation in *Btk*−/− BMMCs was compensated for in DKO cells (compare lanes 7/8 and 17/18; Fig. 4a).

Next, we aimed to corroborate this signalling analysis by the pharmacological approach using Ibrutinib, which qualitatively recapitulated the data obtained by studying the four different BMMC genotypes: (i) P-ERK1/2 was significantly reduced in suboptimally stimulated Ibrutinib-treated WT cells, whereas no such effect was observed in Ibrutinib-treated *Ship1*−/− BMMCs; (ii) enhanced phosphorylation of p38, IKKα/β, and IκBα in *Ship1*−/− cells in response to suboptimal Ag concentration were reduced by Ibrutinib to levels found in WT BMMCs; and (iii) augmented PKB phosphorylation in Ag-stimulated *Ship1*−/− BMMCs remained unchanged in the presence of Ibrutinib (Fig. 4b). Thus, our data indicate differential dependencies and co-dependencies on BTK and/or SHIP1 for different signalling pathways downstream of the FcεRI. Again, patterns of dependence appear to be dictated by the Ag concentration used for FcεRI crosslinking.

### ERK1/2 activation by suboptimal antigen concentrations is dependent on a BTK/PLCγ/DAG module

**Figure 5:**
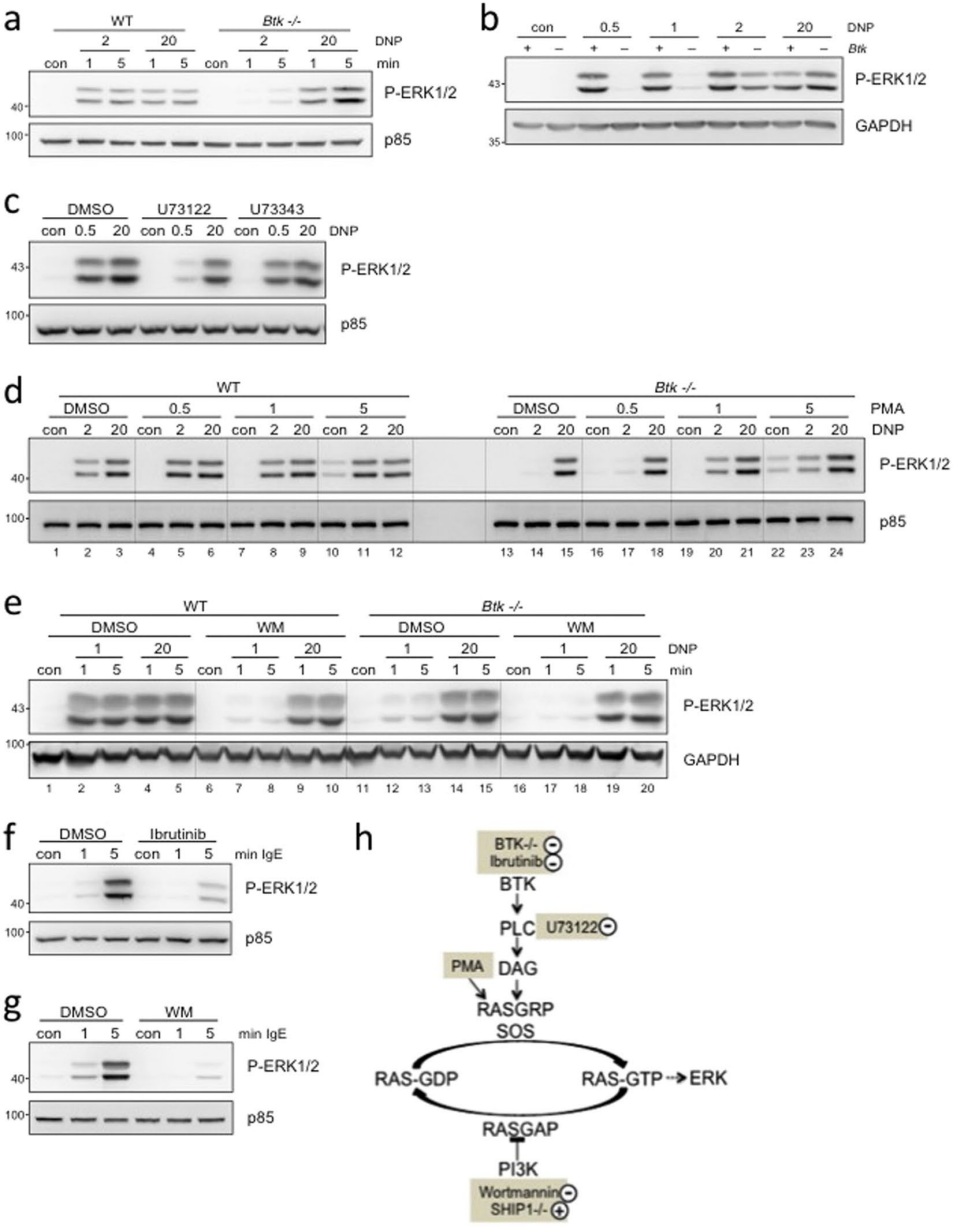
BTK exerts an activating and PI3K a permissive role on ERK1/2 activation in MCs stimulated by suboptimal FcεRI crosslinking. (**a**,**b**) WT (+) and *Btk*−/− (−) PMCs were left untreated (con) or stimulated for 1 and 5 min or (**b**) for 5 min with the indicated amounts of Ag (DNP-HSA [DNP]). (**c**) WT BMMCs were pre-treated for 30 min with 1 µM of the PLC-inhibitor U73122, its inactive compound U73343, or their vehicle (DMSO) and subsequently stimulated for 1 min with suboptimal (0.5 ng/ml) and optimal (20 ng/ml) concentrations of Ag. (**d**) WT and *Btk*−/− BMMCs were left untreated (con) or either stimulated for 1 min in parallel with 2 and 20 ng/ml Ag and increasing amounts of PMA (ng/ml) or vehicle (DMSO). Between lanes 12 and 13, no lysate was applied. (**e**) WT and *Btk*−/− BMMCs were pre-treated for 20 min with 100 nM Wortmannin (WM) before stimulation with 1 and 20 ng/ml Ag for 1 and 5 min. (**f**,**g**) WT BMMCs were pre-treated for 30 min with 0.3 µM Ibrutinib (**f**) or 20 min with 100 nM Wortmannin (**g**) before stimulation for 1 and 5 min with 2 µg/ml IgE (SPE-7). (**a**–**g**) Whole cell-lysates were subjected to immunoblot analysis with the respective antibodies against P-PKB (Ser473), P-ERK1/2 (Thr202/Tyr204), and p85 or GAPDH as loading controls. Comparable results were obtained in at least three experiments with different PMC or BMMC cultures. Fine vertical lines were inserted to allow for better discrimination of cells/conditions. (**h**) Model integrating the positive and negative effects of BTK and PI3K, respectively, on activation of ERK1/2. KO phenotypes and pharmacological activators/inhibitors are highlighted in fawn.

Amongst the studied phosphorylation events, in particular ERK1/2 phosphorylation caught our attention since it hardly showed differences between suboptimal and optimal Ag stimulation of WT and *Ship1*−/− BMMCs. All the more it was interesting that BTK deficiency predominantly impacted on suboptimal stimulation, which was compensated for by additional SHIP1 deficiency, indicating functional interplay between BTK- and SHIP1-controlled signals in regulating ERK1/2 phosphorylation. Such response pattern is reminiscent of a conversion of an analogous input to a digital output as reported for activation of the small GTPase RAS by the cooperation between the different exchange factors, SOS and RASGRP^29^. Hence, we analysed this regulation in more detail and asked first, whether this type of regulation was specifically found in BMMCs or could also be observed in another MC type as a more general mechanism. Therefore, peritoneal MCs (PMCs) from WT and *Btk*−/− mice were investigated. Activation of ERK1/2 in PMCs was also particularly dependent on BTK after suboptimal Ag stimulation (Fig. 5a). Though such qualitative behaviour could be shown for all WT and *Btk*−/− BMMCs and PMCs in the course of our study, the Ag concentration required for demonstration and analysis of this switch-point of analogous-to-digital conversion varied slightly between different MC cultures. An example with a switch-point Ag concentration between 0.5 and 1 ng/ml Ag is shown in Fig. 5b. All Western blot analyses of this study were preceded by determining the switch-point Ag concentration of the respective MC culture, which usually was 1 or 2 ng/ml (rarely 0.5 ng/ml).

With respect to the above-mentioned analogous-to-digital conversion in RAS activation, RASGRPs as DAG- and PKC-dependent enzymes were found to jump-start SOS activation by enabling the establishment of an allosteric, positive feedback loop resulting in RAS and downstream MAPK pathway activation^30^. Since BTK controls PLCγ and hence DAG production, we hypothesised that blocking PLCγ activity might copy the respective BTK-deficient phenotype. Moreover, substituting DAG (by means of its structural analogue PMA) should recover a WT-like phenotype in *Btk*−/− cells. To address the role of PLCγ in Ag-triggered ERK1/2 phosphorylation, the PLCγ inhibitor U73122 and its inactive control compound U73343 were used. IgE-preloaded WT BMMCs were pretreated with DMSO (vehicle control), U73122, or U73343 (control substance) and subsequently stimulated with a suboptimal and an optimal dose of Ag. As shown in Fig. 5c, stimulation with suboptimal and optimal Ag concentrations resulted in comparable ERK1/2 phosphorylation in DMSO- and U73343-pretreated WT cells. In contrast, U73122-mediated inhibition of PLCγ resulted in reduced ERK1/2 phosphorylation, primarily in suboptimally stimulated cells. Thus, with respect to the Ag-triggered phosphorylation of ERK1/2, U73122-treated WT BMMCs reacted in a qualitatively comparable fashion to *Btk*−/− BMMCs (compare Figs 5c and 4). Next, we assessed if the deficit in ERK1/2 phosphorylation in suboptimally stimulated *Btk*−/− BMMCs could be due to insufficient generation of DAG under these conditions. For this reason, WT and *Btk*−/− BMMCs were left unstimulated or stimulated with a combination of DMSO or different PMA concentrations plus Ag (suboptimal or optimal concentration). Subsequently, phosphorylation of ERK1/2 was analysed. Of importance, the relevant PMA concentration (1 µM) did not induce ERK1/2 phosphorylation by itself (Fig. 5d). Interestingly, the combination of PMA plus suboptimal Ag concentration was able to compensate for the lack of BTK in *Btk*−/− BMMCs (Fig. 5d), indicating the importance of a BTK-PLCγ-DAG-ERK1/2 axis, particularly in MCs stimulated by suboptimal Ag concentrations.

It was puzzling that both BTK and PLCγ were not dependent on PI3K (Fig. 1), however increased PI3K pathway activation in DKO BMMCs was able to compensate for BTK deficiency concerning suboptimal ERK1/2 phosphorylation (Fig. 4). In this respect, a permissive role of PI3K for RAS activation by restricting the activity of the negative regulator of RAS, RASGAP1 was identified^31^ (Fig. 5h). This suggested that inhibition of PI3K in WT BMMCs should result in reduced ERK1/2 phosphorylation in suboptimally stimulated cells. Indeed, whereas ERK1/2 phosphorylation in response to optimal Ag concentration remained mostly unaffected by PI3K inhibition and/or BTK deficiency (compare lanes 4/5, 9/10, 14/15, and 19/20), ERK1/2 phosphorylation by suboptimal FcεRI crosslinking was dependent on both PI3K and BTK (compare lanes 2/3, 7/8, 12/13, and 17/18; Fig. 5e). Thus, our data suggest that BTK and PI3K independently control ERK1/2 activation in response to suboptimal Ag concentrations. Since we have previously shown that cytokinergic IgE is able to stimulate marked ERK1/2 phosphorylation^12,13^, we finally aimed to corroborate BTK and PI3K dependence of ERK1/2 phosphorylation under such weak crosslinking condition. WT BMMCs were pre-treated with DMSO (vehicle), BTK inhibitor Ibrutinib (Fig. 5f) or PI3K inhibitor Wortmannin (Fig. 5g), left unstimulated or stimulated with IgE, and phosphorylation of ERK1/2 was measured. Indeed, pharmacological inhibition of both BTK (Fig. 5f) and PI3K (Fig. 5g) resulted in almost complete suppression of IgE-induced ERK1/2 phosphorylation. In conclusion, BTK and PI3K independent from each other regulate the analogous-to-digital switch of ERK1/2 phosphorylation in BMMCs activated by suboptimal FcεRI crosslinking (Fig. 5h).

## Discussion

The opposing phenotypes of Ag-stimulated *Btk*−/− and *Ship1*−/− BMMCs with respect to Ca^2+^ mobilisation, degranulation and cytokine production promoted the idea of BTK being substantial for the SHIP1-deficient phenotype^8–10,14–17^. This assumption was put forth even more since BTK activation was described as PIP_3_-dependent^4,5^ and *Ship1*−/− BMMCs upon stimulation generated high amounts of PIP_3_^11,32^. Therefore, our initial finding of unaltered, Ag-triggered BTK phosphorylation at Y551 and Y223 in the presence of the PI3K inhibitor Wortmannin was rather unexpected. On the other side, also no negative influence on BTK activation of PI3K deficiency was observed in B-lymphocytes^19^. Moreover, PLCγ phosphorylation was demonstrated in Ag-triggered MCs to be both BTK-dependent and PI3K-independent^23^ (this publication), indicating that BTK activation is not necessarily dependent on PI3K in every signalling situation. Nevertheless, the question remains of how BTK can be activated in a largely PI3K-independent manner. Particular compelling in this respect is the recent finding that BTK can be activated by soluble inositol hexakisphosphate (IP_6_)^33^. IP_6_ induced transient BTK dimerization via the PH-TH domains and thus promoted transphosphorylation of the kinase domains even in the absence of a membrane^33^. Of note, IP_6_-BTK interaction is independent of the PIP_3_-binding pocket of the BTK PH-domain^33^. Moreover, generation of IP_6_ emanates from the production of IP_3_, which in MCs is catalysed by PLCγ in a PI3K-independent manner, potentially constituting a BTK-driven positive feedback loop^33^. Those data and our thoroughly controlled results show that activation/phosphorylation of BTK can be independent of PI3K; this, most likely, is dependent on cell type, stimulus, and environmental factors^34^.

**Figure 6:**
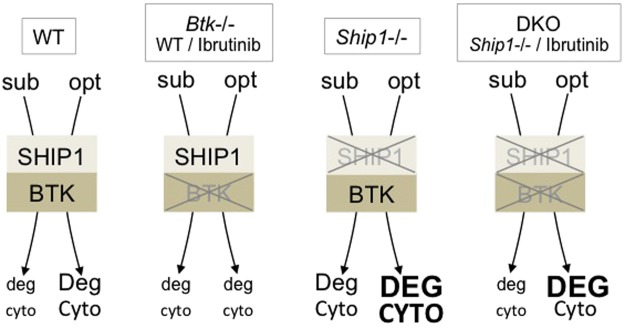
Effects of BTK- and SHIP1-deficiency on Ag-triggered effector functions. WT, *Btk*−/− (or WT cells + Ibrutinib), *Ship1*−/−, and DKO MCs (or *Ship1*−/− cells + Ibrutinib) are opposed to each other in an exemplary manner. Extent of FcεRI crosslinking is specified as suboptimal (sub) or optimal (opt). The level of induction of the analysed effector functions, degranulation and cytokine production, is represented by letter size (deg < Deg < DEG; cyto < Cyto < CYTO).

To elucidate the importance of BTK for the manifestation of the hyperactive SHIP1-deficient phenotype, *Btk*−/− and *Ship1*−/− mice were crossed and eventually, WT, *Btk*−/−, *Ship1*−/−, and DKO BMMCs were differentiated and analysed. Our experiments revealed that BTK-dependence of the SHIP1-deficient phenotype varied depending on Ag concentration used and endpoint analysed (Fig. 6). Whereas degranulation and proinflammatory cytokine production in *Ship1*−/− cells in response to a suboptimal Ag concentration were strictly controlled by BTK, responses to optimal Ag concentrations were less BTK-dependent. There was, however, a marked difference between the effects on degranulation and on cytokine production after optimal Ag stimulation; combined deficiency of BTK and SHIP1 in comparison to single SHIP1 deficiency did only weakly attenuate Ag-triggered degranulation, whereas cytokine production was reduced to WT levels (Fig. 6). Use of the inhibitor Ibrutinib, which covalently binds to Cys481 of the BTK kinase domain and inhibits other TEC family kinases as well^35,36^, revealed comparable activation patterns (Fig. 6). Thus, at first sight, a compensating function of other TEC family kinases expressed in BMMCs (ITK and TEC^37^) might be excluded. However, an extended titration analysis in WT and *Ship1*−/− BMMCs revealed that complete inhibition of degranulation in optimally stimulated *Ship1*−/− BMMCs required about 30-times higher concentrations of Ibrutinib compared to WT cells as well as suboptimally stimulated SHIP1-deficient cells (Supplementary Fig. S3). This suggests that, at least in the absence of BTK, ITK and/or TEC in a compensating manner are activated stronger in optimally stimulated *Ship1*−/− BMMCs, which might contribute to the attenuated effect of BTK deficiency in the *Ship1*−/− background. Interestingly, the PH-domain of ITK was reported to synergistically bind to PIP_3_ and the Ca^2+^-binding protein Calmodulin allowing for positive feedback potentiation of ITK-mediated Ca^2+^ signalling. This feature was not observed for BTK^38^. Activation of additional BTK/ITK/TEC-independent signalling elements in *Ship1*−/− and dko BMMCs cannot be excluded and elaborated transcriptome and proteome analyses should allow to address this question in the future.

While most of the signalling elements studied, such as PLCγ1, IKKα/β, IκBα, p38, and PKB were significantly less phosphorylated/activated in WT BMMCs stimulated with suboptimal compared to optimal Ag concentrations, there was almost no difference in ERK1/2 phosphorylation. Moreover, BTK-deficiency impacted ERK1/2 only after suboptimal stimulation, which was counterbalanced by additional SHIP1-deficiency. This behaviour reminded of an analogous-to-digital switch, where graded inputs are converted into switch-like responses. Such systems usually contain positive feedback and/or double-negative feedback loops^39^. GDP/GTP exchange at the small GTPase RAS is critical for the activation of the RAF-MEK-ERK-containing MAPK pathway. GDP/GTP exchange can be catalysed by two different types of guanine nucleotide exchange factors, the ubiquitously expressed SOS and the differentially expressed members of the RASGRP family. MCs express RASGRP1 and 4^40,41^. SOS not only generates RAS-GTP, but also contains an allosteric RAS-GTP binding pocket, which promotes positive feedback activation after RAS-GTP binding^42^. RASGRPs have been shown to provide RAS-GTP to jump-start SOS activation^29^. This mechanism was suggested to be crucial for ample RAS/MAPK activation in response to physiologically low concentrations of stimuli. Importantly, combined action of RASGRP and SOS can remodel analogous input to digital output^30^. Ag-stimulated ERK1/2 phosphorylation was of comparable strength independent of the Ag concentration used, meaning that a graded input was converted to a digital output. Our comparison between WT and *Btk*−/− BMMCs indicated that this analogous-to-digital switch was disconnected in the absence of BTK (Fig. 5h). BTK is crucial for the activation of PLCγ, which produces the 2^nd^ messengers IP_3_ and DAG. RASGRP and PKC need DAG for activation and membrane recruitment; moreover RASGRP needs to be phosphorylated by PKC^43,44^. Fitting, pharmacological inhibition of PLCγ in WT BMMCs phenocopied *Btk*−/− cells with respect to attenuated ERK1/2 phosphorylation in suboptimally stimulated cells. Moreover, addition of PMA enhanced ERK1/2 phosphorylation in *Btk*−/− BMMCs in response to suboptimal Ag, whereas no effect of PMA was observed after optimal stimulation (Fig. 5h). A complementary possibility might be DAG/PKC-mediated phosphorylation of the RAF kinase inhibitory protein RKIP, which upon its phosphorylation would dissociate from RAF allowing activation of the latter^45^. According to Rubio and Wetzker^31^, PI3K is not directly involved in RAS activation, however, it plays a permissive role by restricting the activity of the negative RAS regulator, RASGAP1. Interestingly, BTK/SHIP1 codeficiency resulted in normalised P-ERK1/2 in response to suboptimal Ag stimulation, suggesting that enhanced activation of the PI3K pathway in the absence of SHIP1 allows for stronger activation of RAS due to attenuation of RASGAP1. In accordance, inhibition of PI3K by Wortmannin caused decreased P-ERK1/2 in suboptimally, but not in optimally Ag-stimulated WT BMMCs (Fig. 5h). Since we did not observe prominent PI3K-dependence of BTK or PLCγ activation, regulation by PI3K of the RAS activation/deactivation cycle on the level of the GTPase-activating protein seems likely. Thus, both positive feedback and double-negative feedback loops, as referred to by Ferrell^39^, appear to be realised in the process of Ag-induced ERK1/2 activation and hence are particularly sensitive to BTK, PLCγ, and PI3K action. Interestingly, the RAS-dependent MAPK pathway appears to be privileged as a signalling pathway allowing for an analogous-to-digital switch.

In summary, our genetic and pharmacological study demonstrates the especial prominence of BTK for the SHIP1-deficient phenotype of MCs activated via the FcεRI by suboptimal Ag concentrations. Moreover, BTK activity appears to contribute to the outcome of SHIP1 deficiency after optimal-to-supraoptimal FcεRI crosslinking, though with less consequence. With respect to signal transduction, in particular activation of the MAPK pathway is dependent on BTK, which allows for an analog-to-digital switch resulting in full activation of ERK1/2 already at suboptimal Ag concentrations. Our data suggest that under non-suboptimal conditions, reduced activation/expression of SHIP1 and BTK can be compensated/corrected by pharmacological inhibition of BTK and SHIP1, respectively.

## Methods

### Animals and cell culture

The following mutant mice were used: *Btk*−/− mice^46^ and *Ship1*−/− mice (*Inpp5d*−/−)^47^. Mating of these mice resulted in *Btk*+/− *Ship1*+/− F1 progeny. F1 mice were mated to obtain WT, *Btk*−/−, *Ship1*−/−, and *Btk*−/−*Ship1*−/− mice (referred to as DKO). All mice were on a mixed C57BL/6 × 129/Sv background. Experiments were performed in accordance with German legislation governing animal studies and following the Principles of Laboratory Animal Care. Mice are held in the Institute of Laboratory Animal Science, Medical Faculty of RWTH Aachen University. The Institute holds a license for husbandry and breeding of laboratory animals from the Veterinary Office of the Städteregion Aachen (Administrative District). The Institute follows a Quality Management System, which is certified according to DIN ISO 9001/2008. Every step in this project involving mice was reviewed by the animal welfare officer. All experiments were approved by the Landesamt für Natur, Umwelt und Verbraucherschutz NRW (LANUV), Recklinghausen (AZ 84-02.04.2016.A496).

For the generation of BMMCs according to the procedures by Razin *et al*.^48^, bone marrow cells (1 × 10^6^/ml) from 6- to 8-week old mice were cultured at 37 °C and 5% CO_2_ as single cell suspensions in growth medium (RPMI 1640 medium containing 15–20% FCS, 2% X63Ag8-653-conditioned medium (source of IL-3^49^), 2 mM L-glutamine, 10 mM HEPES, 1 × 10^−5^ M 2-mercaptoethanol, 100 units/ml penicillin, and 0.1 mg/ml streptomycin). At weekly intervals, the non-adherent cells were reseeded at 1 × 10^6^ cells/ml in fresh medium. By 4–6 weeks in culture, greater than 95% of the living cells were positive for KIT and FcεRI as assessed by PE-labelled anti-KIT (#553869; BD Pharmingen) and FITC-labelled anti-FcεRI alpha antibodies (#11-5898; eBioscience), respectively. Expression of ST2 was detected by FITC-conjugated anti-mouse T1/ST2 (#101001F; mdbioproducts) and of CD13 by PE-conjugated rat anti-mouse CD13 (#558745; BD Pharmingen). Peritoneal MCs (PMCs) were prepared and cultivated using IL-3 and SCF according to Meurer *et al*.^50^.

### Antibodies and reagents

Antibodies directed against P-PKB (Ser473; #4051), P-ERK1/2 (Thr202/Tyr204, #4370), P-IKKα/β (Ser176/Ser177, #2078), P-IκBα (Ser32, #2859), P-LAT (Tyr191, #3584), P-p38 (Thr180/Tyr182, #9216), P-PLCγ2 (Tyr759, #3874), and PI3K p85 (#4292) were purchased at Cell Signaling Technologies. Anti-GAPDH (sc-32233), anti-BTK (sc-1696), and anti-P-PLCγ1 (Tyr783; sc-12943) antibodies were from Santa Cruz Biotechnology and anti-PLCγ1 (#2112-1) antibody wase obtained from Epitomics. The antibody against P-BTK (Tyr551; #MAB7659) was purchased at R&D Systems. The following inhibitors were used: for BTK, Ibrutinib (PCI-32765, A11020, Adooq Bioscience), for PLC, U73122 (BML-ST391, Enzo) as well as its inactive compound U73343 (BML-ST392, Enzo), and for PI3K, Wortmannin (#681676, Merck Millipore). Dinitrophenyl (DNP)-human serum albumin (HSA) containing 30–40 mol DNP per mol albumin (A6661) and anti-DNP-specific monoclonal IgE (clone SPE-7, D8407) were purchased at Sigma-Aldrich. DMSO (A3672) was obtained from PanReac Applichem and Phorbol-12-myristate-13-acetate (PMA, #524400) from Merck Millipore.

### Stimulation of mast cells

MCs were pre-loaded overnight with 0.15 µg/ml IgE in starvation medium (without IL-3) or in growth medium (without SCF) for BMMCs or PMCs, respectively. The next day, the cells were washed with PBS and resuspended at a density of 1–2 × 10^6^ cells/ml in stimulation medium (RPMI 1640, 10 mM HEPES, 0.1% BSA) for signal transduction studies and cytokine production or in Tyrode’s buffer (130 mM NaCl, 5 mM KCl, 1.4 mM CaCl_2_, 1 mM MgCl_2_, 5.6 mM glucose, and 0.1% BSA in 10 mM HEPES, pH 7.4) for degranulation studies and adapted to 37 °C for 20 min. If necessary, the cells were treated at 37 °C with the respective inhibitor for the mentioned times before stimulation or were directly stimulated 20 min for degranulation, 4 h for the production of cytokines (IL-6 and TNF-α), or as indicated for the signal transduction analyses.

### Degranulation

For degranulation studies, the percentage of degranulation was determined by measuring the release of β-hexosaminidase^51^.

### SDS-PAGE & Western Blot analysis

Following stimulation, cells were pelleted and the pellets were solubilised at 4 °C in phosphorylation solubilisation buffer^52^ containing 0.5% IGEPAL and 0.5% Na-deoxycholate. To prevent degradation during lysis, the PMC pellets were directly lysed in 95 °C hot 1 x Laemmli buffer for 10 min. Whole-cell lysates of BMMCs, after centrifugation at 4 °C with 16,000 × g for 15 min, were subjected to SDS-10%-PAGE. For Western Blot analysis, the proteins were blotted for 1–2 hours with 350 mA onto a PVDF membrane (#10600023; GE Healthcare Life Sciences) using a 25 mM Tris, 192 mM glycine, and 20% methanol solution. The membrane was blocked with 2% w/v milk powder in wash buffer (PBS 0.1% Tween) for 1 h at RT before the incubation with the respective primary antibody overnight. Particularly for detection of P-BTK (Tyr551), the PVDF membrane was dried overnight after protein transfer, reactivated by methanol treatment, and blocked with 10% w/v BSA in wash buffer before incubation with primary antibody overnight at 4 °C. The next day, following incubation with HRP-coupled secondary antibody, the membrane was developed with enhanced chemiluminescence solution using a LAS 4000 (GE Healthcare Life Sciences).

### Cytokine ELISAs

The supernatants of stimulated BMMCs that were centrifuged for 10 min at 1,000 × g, were used for the detection of secreted cytokines. Mouse IL-6 (BD Pharmingen) and mouse TNF-α ELISAs (R&D Systems) were performed according to the manufacturer’s instructions. Concentrations of cytokines varied between experiments and/or cell cultures due to age of the cells. Qualitative differences or similarities between the genotypes, however, were consistent throughout the study.

### Calcium measurement

IgE-preloaded BMMCs were resuspended at 1 × 10^7^ cells/ml in RPMI 1640 containing 1% FCS and stained with 50 µM Indo-1 AM (I1226; Thermo Fisher Scientific) and 0.5% pluronic F-127 (P3000MP; Thermo Fisher Scientific) for 45 min at 37 °C. Afterwards, cells were resuspended in RPMI/1% FCS and analysed with the indicated stimulations using a LSR II (BD). FACS profiles were converted to line graph data using the FlowJo analysis software (Treestar).

### Statistical analysis

Statistical data were analysed from n independent experiments (with n indicated in the respective figure legend) using PRISM version 7.0c (GraphPad software, Inc.). P-values were calculated by a two-tailed Student’s t-test for WT vs. DKO cells and by a one-tailed Student’s t-test for BTK−/− or SHIP1−/− vs. DKO cells. P-values smaller than 0.05 with (*)p < 0.05, (**)p < 0.005, and (***)p < 0.0005 were considered statistically significant and p-values bigger than 0.05 were non significant (n.s.). No adjustment for multiple testing was performed since all analyses were explorative.

## Electronic supplementary material

Supplementary Information

## Data Availability

The datasets generated during and/or analysed during the current study are available from the corresponding author on reasonable request.
