# Supplementary material for: Stimulus strength determines the BTK-dependence of the SHIP1-deficient phenotype in IgE/antigen-triggered mast cells
Source: Sci Rep. 2018 Oct 19;8:15467. doi: 10.1038/s41598-018-33769-1 (PMC6195619; doi:10.1038/s41598-018-33769-1)
Supplement: Supplementary file 1 — Supplementary Information [file 41598_2018_33769_MOESM1_ESM.pdf]

Supplementary information

Stimulus strength determines the BTK-dependence of the SHIP1-deficient phenotype in IgE/antigen-triggered mast cells

Carolin N. Zorn, Anne Simonowski, and Michael Huber[1]

Institute of Biochemistry and Molecular Immunology, University Clinic, RWTH Aachen University, Pauwelsstraße 30, 52074 Aachen, Germany

[1] Corresponding author: Dr. Michael Huber (PhD)

Institute of Biochemistry and Molecular Immunology

RWTH Aachen University

Pauwelsstr. 30, 52074 Aachen, Germany

Tel: ++49-241-8088830; Fax: ++49-241-8082428

E-mail: mhuber@ukaachen.de

Supplementary Figure S1

Illustration of the sequence homology between the TEC family kinases BTK, TEC, ITK, and TXK.

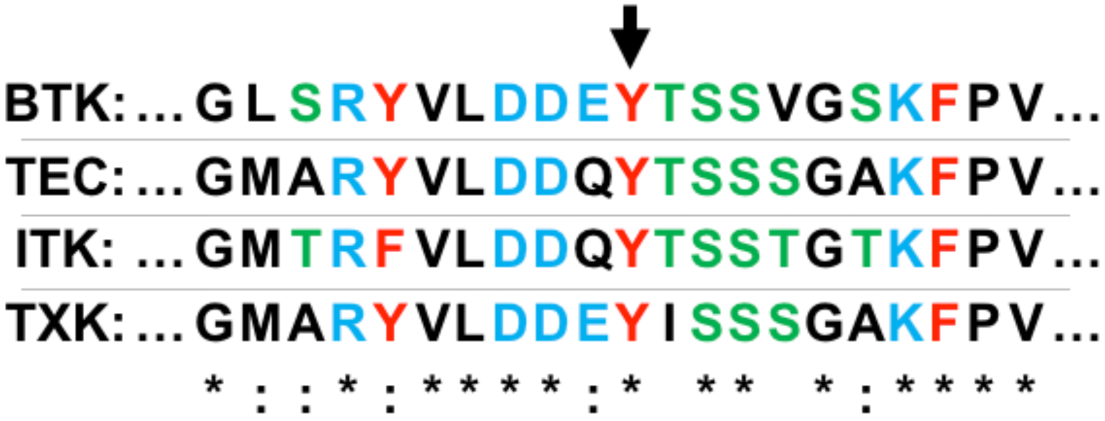

Amino acid sequences of the murine TEC family kinases BTK, TEC, ITK, and TXK were aligned using the online tool Clustal Omega (www.clustal.org/omega/). The arrow depicts the Y residues in the respective kinase domains necessary for activation of the TEC family kinases (BTK, Y551; TEC, Y518; ITK, Y517; TXK, Y420). Shown are ten amino acids N-terminal and C-terminal of this activating Y residue. Aromatic amino acids are coloured in red, charged amino acids in blue, and S/T residues in green. The degree of conservation is indicated by the following signs (gradation: ***** > **:** > blank).

Supplementary Figure S2

Comparable expression of mast cell surface markers on WT, Btk-/-, Ship1-/-, and DKO BMMCs.

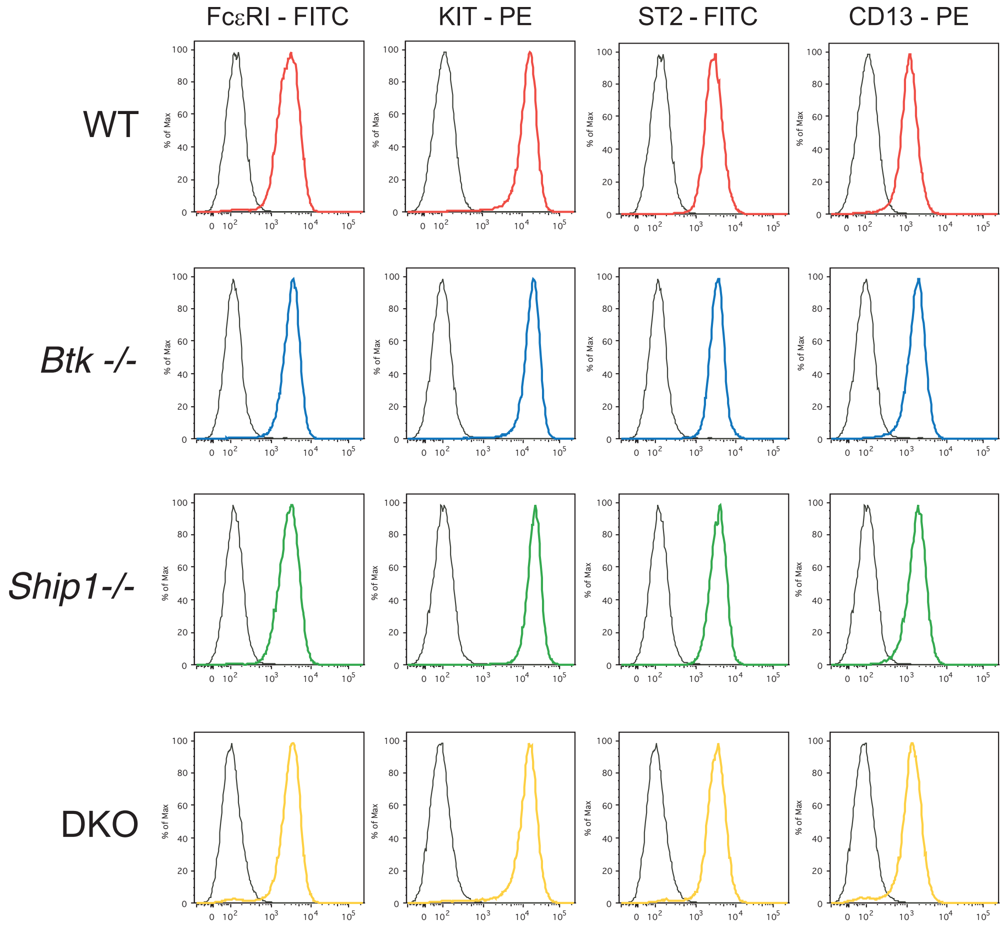

WT (red), *Btk-/-* (blue), *Ship1-/-* (green), and DKO (yellow) BMMCs were analysed for the differentiation state with FITC-conjugated anti-FcεRI alpha, PE-conjugated anti-KIT, FITC-conjugated anti-ST2, and PE-conjugated anti-CD13 antibodies in a FACS Canto II (BD Biosciences). Comparable results were obtained with cells from different MC cultures. (n = 4)

Supplementary Figure S3

Complete inhibition of degranulation in Ship1-/- BMMCs requires higher concentrations of Ibrutinib than in WT BMMCs.

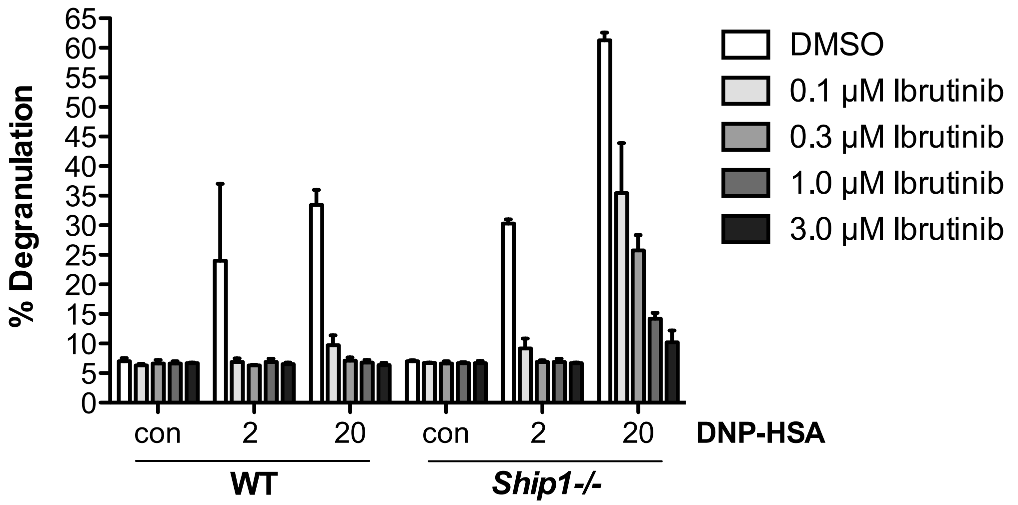

WT and *Ship1-/-* BMMCs were preloaded overnight with 0.15 µg/ml DNP-specific monoclonal IgE (SPE-7). The cells were pretreated with 0.1, 0.3, 1.0, and 3.0 µM Ibrutinib for 30 min at 37 °C before stimulation. Following, the cells were left untreated (con) or stimulated with 2 and 20 ng/ml Ag (DNP-HSA) for degranulation and the amount of released β–hexosaminidase was determined. Each bar is the mean of triplicates ± SD; n = 2.
